# Community Structure and In Situ Activity of Nitrifying Bacteria in *Phragmites* Root-Associated Biofilms

**DOI:** 10.1264/jsme2.ME11314

**Published:** 2012-03-23

**Authors:** Satoshi Okabe, Yoshiyuki Nakamura, Hisashi Satoh

**Affiliations:** 1 Division of Environmental Engineering, Faculty of Engineering, Hokkaido University, North-13, West-8, Kita-ku, Sapporo Hokkaido 060–8628, Japan

**Keywords:** *Phragmites*, root biofilms, oxygen release, AOB, NOB

## Abstract

The amount of oxygen released by *Phragmites* roots and the community structure and in situ activity of nitrifying bacteria in the root biofilms were analyzed by the combined use of 16S rRNA gene-cloning analysis, quantitative PCR (qPCR) assay and microelectrodes. Axial and radial O_2_ microprofiles were obtained for individual roots of *Phragmites* in a horizontal flow reactor fed with artificial medium continuously. Axial O_2_ profiles revealed that O_2_ was released at a rate of 0.21 μmol O_2_ cm^−2^ (root surface area) h^−1^ only in the apical region (up to ca. 40 mm from the root apex), where there was a high abundance (10^7^ to 10^8^ copies g^−1^ biomass) of *Nitrosomonas*-like AOB and *Nitrospira*-like NOB. This abundance, however, sharply declined to the detection limit at positions more basal than 80 mm. Phylogenetic analysis based on 16S rRNA gene identified strains related to *Nitrosomonas oligotropha* and *Nitrosomonas cryotolerans* as the predominant AOB and strains related to *Nitrospira marina* and *Nitrospira moscoviensis* as the predominant NOB in the root biofilms. Based on radial O_2_ microprofiles, the oxic region only extended about 0.5 mm into the surrounding sediment due to a high rate of O_2_ consumption in the rhizosphere. The net NH_4_^+^ and O_2_ consumption rates in the apical region were higher than those determined at the oxic sediment surface in which the abundance of AOB and NOB was one order of magnitude lower than in the rhizosphere. These results clearly indicated that *Phragmites* root biofilms played an important role in nitrification in the waterlogged anoxic sediment.

Nitrification, the microbially mediated oxidation of NH_4_^+^ to NO_3_^−^, plays a central role in the N-cycle of aquatic environments, especially with respect to the growing eutrophication of aquatic environments. Since waterlogged environments (*e.g.*, river banks and sediments) become anoxic almost immediately beneath the sediment-water interface, nitrification is expected to occur only in narrow surface oxic zones (44). It is, however, recognized that the immediate root surface of emergent plants, such as *Phragmites*, offers a supply of oxygen through leakage from aerenchymatous tissue (3, 6, 10), which may promote nitrification. Oxygen-releasing plants might therefore have a significant impact on nitrogen cycling (*i.e.*, nitrification) in otherwise anoxic sediment layers. Plant-induced nitrification can subsequently lead to enhanced denitrification, thus increasing nitrogen removal from aquatic environments.

Despite the longstanding realization that emergent plant roots release oxygen and affect microbial communities in the immediate rhizosphere, the relationship between oxygen release from roots and structure and in situ activity of nitrifying bacteria in the rhizosphere is not fully understood because of difficulty in the direct measurement of nitrifying activity and oxygen release from plant roots and the limitations of traditional culture-dependent techniques. There appears to have been no published work reporting combined information about the diversity, abundance, and fine-scale distribution of the in situ activity of nitrifying bacteria in the immediate rhizosphere in aquatic sediments. Therefore, it is imperative to directly quantify the available oxygen concentration and the in situ activity and abundance of nitrifying bacteria in the immediate rhizosphere.

Cultivation-independent molecular techniques have provided better understanding of the diversity and distribution of nitrifying bacteria in various aquatic environments, in which nitrifying bacteria were usually found in small numbers (1, 8, 28, 43). Recently, Briones *et al.*(7) analyzed ammonia-oxidizing bacterial (AOB) communities in the rhizosphere of rice by using 16S rRNA gene-based denaturing gradient gel electrophoresis (DGGE) and fluorescence in situ hybridization (FISH) and found that the detection frequencies of *Nitrosomonas* and *Nitrosospira* varied with plant variety. The diversity and abundance of nitrite-oxidizing bacteria (NOB) in freshwater sediments and rhizospheres were, however, neglected in most of the studies. The molecular approach has been applied successfully in the rhizospheres (14, 35, 46) and phytosphere (41, 50) of various plants, demonstrating plant-dependent enrichment or the diversity of microorganisms. In addition, the combination of molecular techniques with microelectrodes has been proven to be a powerful tool to analyze the structure and in situ activity of microbial communities in freshwater sediments (1, 28, 44), biofilms (21, 31–34, 45), microbial granules (43), and rhizospheres (7).

The goal of this study was to investigate the relationship between oxygen release from the *Phragmites* roots and structure and in situ activity of nitrifying bacterial populations in the root biofilms. In this study, we directly determined the microprofiles of substrates (O_2_, NH_4_^+^, NO_3_^−^ and pH) in the rhizosphere of *Phragmites* by using microelectrodes to quantify radial oxygen release and the in situ activity of nitrifying bacteria. We also applied 16S rRNA gene-based techniques (16S rRNA gene-cloning and quantitative PCR (qPCR)) to analyze the community structure and abundance of nitrifying bacteria (both AOB and NOB) in the root biofilms.

## Materials and Methods

### Plant material and reactors

**Fig. 1:**
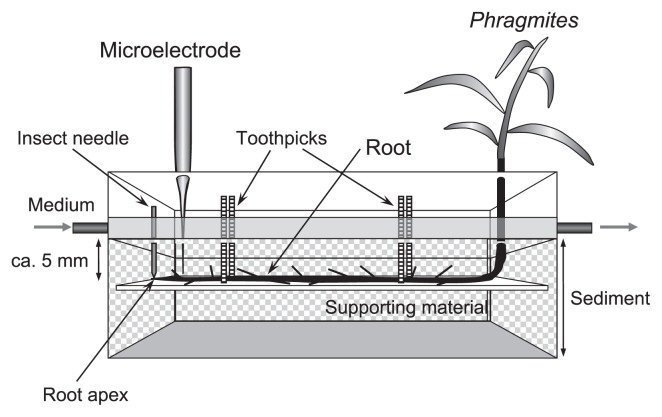
Schematic drawing of a horizontal flow reactor for the culture of *Phragmites* and the measurements of substrate concentration profiles in the rhizosphere.

*Phragmites communis* with horizontal rhizomes and roots was carefully collected from the Sosei River, Sapporo, Japan, which receives treated domestic wastewater. A horizontal flow reactor (20 [L]×5 [W]×5 [H] cm) was constructed using acrylic boards. Total working volume was 400 cm^3^. Approximately 20 cm tall *Phragmites* with roots 10–15 cm long were selected, transferred, and positioned in the reactor, as shown in Fig. 1. Some roots were gently but firmly fixed onto supporting material using toothpicks. The roots were positioned horizontally ca. 5 mm below the surface of the sediment and close to the acrylic sidewall for microelectrode measurements. The positions of the root apex were marked with inset needles. The reactor was carefully filled with fine silt (after passing through a 2 mm sieve), which was taken from the sampling site of *Phragmites communis*.

For the culture of *Phragmites* and the measurements of substrate concentration profiles in the rhizosphere within the sediment, a synthetic medium was continuously fed into the reactors at a flow rate of ca. 40 mL h^−1^. The synthetic medium consisted of MgSO_4_·7H_2_O (40 μM), CaCO_3_ (100 μM), K_2_HPO_4_ (25 μM), EDTA (135 μM), NaNO_3_ (100 μM), NaNO_2_ (50 μM), NH_4_Cl (50 μM), FeSO_4_·7H_2_O (25 μM), NaB_4_O_7_ (5.75 μM), MnCl_2_·4H_2_O (1.125 μM), CuSO_4_·5H_2_O (0.075 μM), Na_2_MoO_4_·2H_2_O (0.025 μM), and yeast extract (5 mg L^−1^). The pH was adjusted to 7. The *Phragmites* was cultured for 8 weeks of 12-h light and dark cycles in the laboratory at 20°C. During the light cycle, plants received 1,450 μmol photons m^−2^ s^−1^. An identical reactor without a plant was operated in parallel as a non-rhizosphere control.

### Microelectrode measurements

Steady-state concentration profiles of O_2_, NH_4_^+^, NO_3_^−^, and pH in the rhizosphere were measured using microelectrodes (6, 40). A Clark-type O_2_ microelectrode was prepared and calibrated as previously described (39). LIX-type microelectrodes for NH_4_^+^, NO_3_^−^, and pH were constructed, calibrated, and used according to the protocol described by Okabe *et al.*(31, 32). The reactor was then acclimated in the light condition for at least a few hours before measurements to ensure that steady-state profiles were obtained.

The microelectrodes that were attached to a motor-driven micromanipulator were positioned manually near the root, and then the micromanipulator was operated in all three directions for fine positioning. We first ensured that the microelectrode tip touched the surface of the root at a given distance from the apex by monitoring changes in the readings carefully (the reading jumped when the microelectrode tip touched the root surface). The position of the microelectrode was defined as the starting point (0, 0) of the microprofiles. The concentration profiles in the rhizosphere were then measured in steps of 50 μm from the root surface to the bulk sediment or the overlaying water. The sediment surface was determined by using a dissection microscope model Stemi 2000 (Carl Zeiss). For radial profile measurements, the microelectrodes were positioned vertically above the root so that the microelectrodes penetrated the center of the roots. Microprofiles were also measured axially along the root surface. To ensure reproducibility of the measurements, the microprofiles were measured again by tracing the original path toward the root surface. Both of the results were usually in very close agreement; therefore, the concentration profile data reported in this paper were obtained as the microelectrodes were moved away from the root surface.

Based on the concentration profiles measured, the total O_2_, NH_4_^+^ and NO_3_^−^ consumption rates in the rhizosphere were calculated using Fick’s first law of diffusion (32). The molecular diffusion coefficients used for the calculations were 2.06×10^−5^ cm^2^ s^−1^ for O_2_, 1.93×10^−5^ cm^2^ s^−1^ for NH_4_^+^, and 1.89×10^−5^ cm^2^ s^−1^ for NO_3_^−^ in water at 20°C (11). The radial oxygen loss (ROL, [mole O_2_ cm^−2^ root surface h^−1^]) from a root to the rhizosphere was calculated from the gradient of the O_2_ microprofile, as described elsewhere (4). The plant oxygen release in the horizontal flow reactor and the field was also estimated as described previously (6). The active root surface area was calculated based on the root diameter and the active root length (*i.e.*, root length over which ROL could be measured). The number of roots per unit sediment surface area was directly measured in the horizontal flow reactor and the field.

### DNA extraction and PCR amplification

After microelectrode measurements, the *Phragmites* were removed from the horizontal flow reactor. The roots were shaken gently to remove soil that loosely adhered to the roots. We found that the root surface was covered with brown thin biofilm (usually about 100-μm thick) (Fig. S1). The biofilm firmly attached to the roots was subjected to the following molecular analyses of microbial community structure.

All selected roots were cut into 5-mm fragments from the root apex with a sterilized razor. The fragments were then grouped into subsamples: 0–5 mm, 5–10 mm, 10–15 mm, 15–20 mm, 20–25 mm, 25–30 mm, 30–35 mm, 35–40 mm, 45–55 mm, 55–65 mm, 65–75 mm, 75–85 mm, and 155–165 mm. Surface sediment samples were also included in the analysis for comparison. Prior to DNA extraction, for each root fragment, 3 g roots with firmly adhering biofilm were placed in 9 mL pure water and vortexed for 3 min. The roots were removed, placed in 9 mL pure water again, and treated in the same way. This treatment was repeated once more. The resulting supernatants of three treatments were combined. Biomass pellets were harvested by centrifugation (10,000×*g*, 30 min) and kept at −80°C until DNA extraction.

Total DNA was extracted from each biomass pellet using a Fast DNA spin kit (MP Biomedicals, Tokyo Japan) as described in the manufacturer’s instructions. The extracted DNA samples (from 0 mm to 85 mm) were combined for the following 16S rRNA gene-cloning analysis for betaproteobacterial ammonia-oxidizing bacteria (AOB), nitrite-oxidizing bacteria (NOB), and ammonia-oxidizing archaea (AOA) communities. To reduce the possible bias caused by PCR amplification, the 16S rRNA gene in the combined subsamples was amplified in triplicate tubes and then combined for the next cloning step. The 16S rRNA gene fragments from the combined total DNA were amplified with Taq DNA polymerase (TaKaRa Bio, Ohtsu, Japan) using previously described protocols and the AOB specific primer set of CTO189f and CTO654r (24), the *Nitrospira*-like NOB specific primer sets of NTSPAf (28)-universal 1492r (51) and Ntspa685 (20) -NTSPAr (28), and the archaeal specific primer set of Arch21F (13) -1492r (51). Comparative investigation of the microbial community other than AOB and NOB in the root biofilm and bulk sediment was conducted using the universal primer set of 8F-1492r (51). All PCRs were performed for 35 cycles using the PCR conditions previously reported for each primer set. PCR products were checked by electrophoresis on a 1% (w/v) agarose gel. In addition, since the archaeal 16S rRNA gene was not amplified from the root biofilms, the archaeal *amoA* gene was also amplified using previously described protocols and the primer set of Arch-AmoAF/Arch-AmoAR (16); however, the archaeal *amoA* gene was also not amplified probably due to low abundance of AOA in the root biofilms.

### Cloning and sequencing of the 16S rRNA gene and phylogenetic analysis

PCR products were ligated into a pCR-XL-TOPO vector and transformed into ONE SHOT *Escherichia coli* cells (TOPO XL PCR cloning; Invitrogen) following the manufacturer’s instructions. Partial sequencing of the 16S rRNA gene inserts (about 470 bp for AOB and 410 bp and 500 bp for *Nitrospira*-like NOB) was performed using an automatic sequencer (ABI Prism 3100 Avant Genetic Analyzer; Applied Biosystems) with a BigDye Terminator Ready Reaction kit (Applied Biosystems). All sequences were checked for chimeric artifacts by the CHECK_CHIMERA program in the Ribosomal Database Project (25) and compared with similar sequences of the reference organisms by a BLAST search (2). Sequence data were aligned with the CLUSTAL W package (49). Clone sequences with 97% or greater similarity were grouped into the same operational taxonomic unit (OTU) by a SIMILARITY_MATRIX program, and their representative sequences were used for phylogenetic analysis. Phylogenetic trees were constructed using the neighbor-joining method (42). Tree topology was also tested using the maximum-parsimony method. Bootstrap resampling analysis of 1,000 replicates was performed to estimate the confidence of the tree topologies.

### Quantification of AOB and NOB by qPCR

Quantitative PCR (qPCR) assays were performed to quantify copy numbers of betaproteobacterial AOB and *Nitrospira*-like and *Nitrobacter*-like NOB-specific 16S rRNA genes in root biofilms at different locations along the root (as mentioned above). Since no PCR products were obtained using the archaeal 16S rRNA gene and archaeal *amoA* gene specific primer sets, their quantification was not performed in this study. The 16S rRNA genes of betaproteobacterial AOB were quantified using the primer set of CTO 189fA/B/C and RT1r and TaqMan probe TMP1, as previously described by Hermansson and Lindgren (18). The primers CTO 189fA/B and CTO 189fC were used at a 2:1 ratio. Standardization was performed with a dilution series (10 to 10^8^ copies per well) of genomic DNA of *Nitrosomonas europaea* (NBRC 14298). The amplification efficiency was calculated to be 87–91% from the equation, ɛ_c_=10^−1/S^−1. The 16S rRNA genes of *Nitrospira*-like NOB were quantified using the primer set of NTSPAf and NTSPAr and TaqMan probe NTSPATaq, as described by Nakamura *et al.*(28). Standardization was performed with a dilution series (10 to 10^7^ copies per well) of the standard vector plasmid carrying ca. 400 bp of 16S rRNA gene of uncultured bacterium A-4 (AF033559) related to Candidatus *Nitrospira defluvii*(28). The amplification efficiency was 91 to 95%.

The *Nitrobacter*-like NOB 16S rRNA gene was quantified as previously described by Degrange and Bardin (12) in a total volume of 25 μL with SYBR Green PCR master mix (PE Applied Biosystems), 300 nM of each of the forward primer FGPS872f and reverse primer FGPS1269r (12), 100 nM of bovine serum albumin (Sigma) and either 0.1 pg sample DNA or 10 to 10^5^ copies per well of the standard genomic DNA of *Nitrobacter winogradskyi* (NBRC 14297). The specificities of PCR products were confirmed by melting curve analysis. qPCR assays for AOB, *Nitrospira*-like NOB, and *Nitrobacter*-like NOB were performed in MicroAmp Optical 96-well reaction plates with an optical cap (PE Applied Biosystems). The template DNA in the reaction mixtures was amplified and monitored with an ABI prism 7000 Sequence Detection System (PE Applied Biosystems). The detection limits for AOB, *Nitrospira*-like NOB and *Nitrobacter*-like NOB in this study were 2.7×10, 1.6×10^2^, and 5.4×10 copies per well, respectively, which corresponded to 6.7×10^4^, 4.0×10^4^, and 1.4×10^4^ copies/cm^3^ when the sample volume and DNA extraction step were taken into account.

### Analytical methods

Total bacterial cell counts in root biofilms taken at different locations along the root (as mentioned above) and in surface sediment samples were also performed after the diluted sediment samples on 0.2-μm-membrane filters were stained with 6-diamidino-2-phenylindole (DAPI). At least 20 replicate analyses were performed for each sample. The NH_4_^+^, NO_2_^−^ and NO_3_^−^ concentrations in the influent and effluent of the reactors were determined using an ion chromatograph (HIC-6A; Shimadzu) equipped with a Shim-pack CS16 column and CS6A. The samples for NH_4_^+^, NO_2_^−^ and NO_3_^−^ were filtered through 0.2-μm pore membrane filters (DISMIC-13CP; Advantec) before analysis. The O_2_ concentration and pH in the overlaying water were determined using an O_2_ and a pH electrode, respectively. Light intensity was measured by a quantum meter (Fujiwara Scientific Company, Tokyo Japan), which senses only in the 400–700 nm region. All measurements were made above the water surface.

### Nucleotide sequence accession numbers

The GenBank/EMBL/DDBJ accession numbers for the 16S rRNA gene sequences of the clones used for phylogenetic tree analysis are AB255039 to AB255048 and AB373105 to AB373109.

## Results

### Water quality in the horizontal flow reactors

The *Phragmites* were cultured for 8 weeks under 12-h light and dark cycles in the laboratory at 20°C. Steady-state conditions were attained after about one month. NH_4_^+^ concentration in the effluent decreased along with pH decline while NO_3_^−^ concentration increased in the horizontal flow reactors with and without *Phragmites*, indicating the occurrence of nitrification. The net NH_4_^+^ consumption rate in the reactor with *Phragmites* was 0.039±0.016 μmol/cm^2^/h (average±SD), which was higher than that obtained in the reactor without the plant (0.020±0.007 μmol/cm^2^/h) (*p*<0.05) (Table 1).

### O_2_ concentration profiles in the rhizosphere

**Fig. 2:**
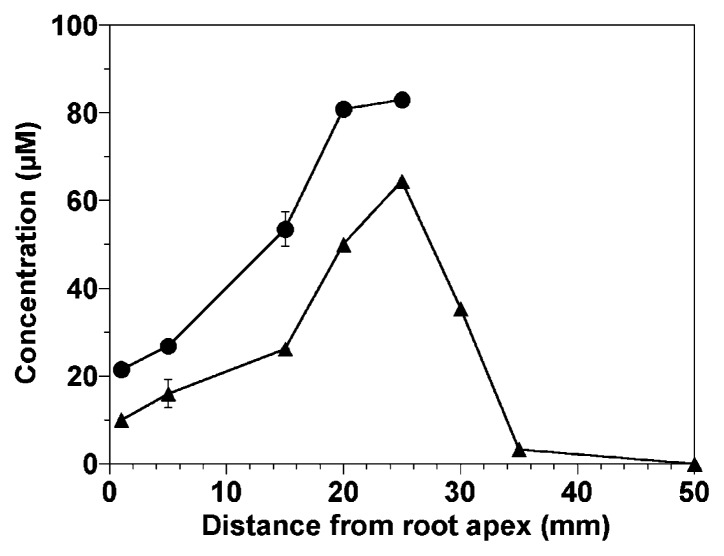
Oxygen concentrations within the cortex (●) and at the root surface (▲) along roots. The roots were immersed in anoxic sediment; the shoot was exposed to air.

Changes in O_2_ concentrations within the cortex and at the root surface with distance along an intact 100-mm long *Phragmites* root are shown in Fig. 2. Microelectrode measurements were made five times, and the average is presented. The cortical oxygen concentration gradually increased toward the root base. In contrast, the O_2_ concentration at the root surface was highest (62 μM) at ca. 25 mm from the root apex and declined markedly at positions more basal than 25 mm, indicating the overall decline in radial oxygen loss (ROL). We could not insert microelectrodes into the root base (more basal than 30 mm from the apex) due to high penetration resistance.

**Fig. 3:**
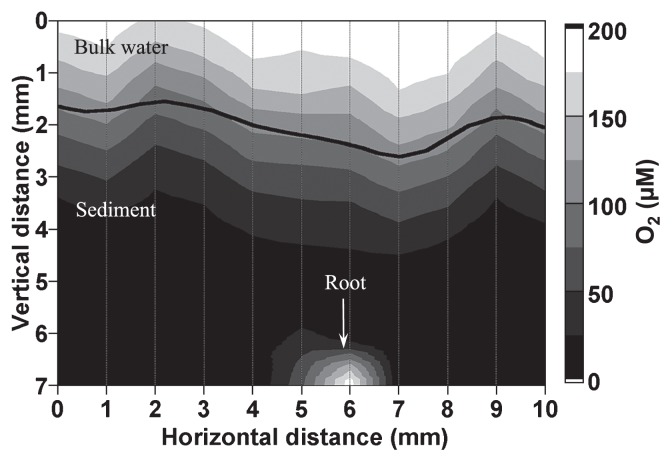
A representative 2-D contour plot of O_2_ concentrations in vertical cross section of the sediment in the reactor. The O_2_ concentrations were measured in light conditions. Numbers in the right margin indicate O_2_ concentrations. The sediment surface, determined by microscopic observation, is indicated by a bold line.

Figure 3 shows a two-dimensional O_2_ concentration profile around the 100-mm long *Phragmites* root, which was immersed about 5 mm below the sediment surface. The root cross-sectional position corresponded to ca. 25 mm from the root apex. The root diameter was approximately 1 mm. O_2_ penetration depth was about 2 mm from the sediment surface, below which anoxic conditions prevailed. More than 150 μM O_2_ was detected inside the root, and O_2_ diffused and radiated out from the root surface, creating an oxic microenvironment around the root.

### Abundance of AOB and NOB along the root

**Fig. 4:**
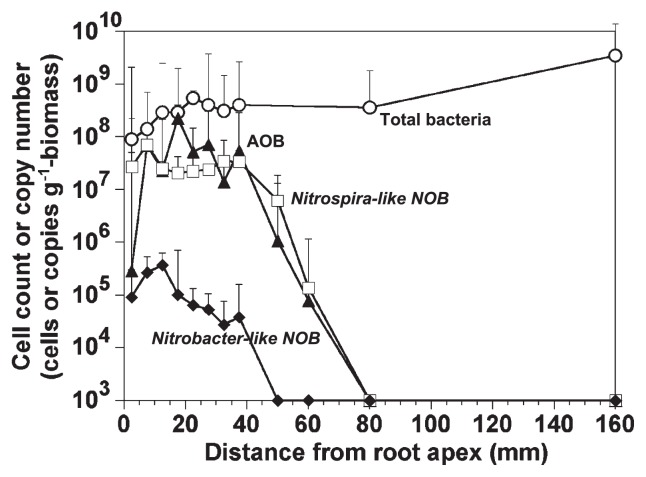
16S rRNA gene copy numbers of AOB in the class of *Betaproteobacteria* (▲), *Nitrospira*-like NOB (□) and *Nitrobacter*-like NOB (◆), and total DAPI count (○) in the root biofilms along roots. The points are mean values ± standard deviations.

Figure 4 indicates axial distributions of the total bacterial cell counts and the specific 16S rRNA gene copy numbers of the betaproteobacterial AOB and *Nitrospira*- and *Nitrobacter*-like NOB in the biofilm sampled along the root. The total bacterial cell numbers gradually increased from 8.9±2.5×10^7^ cells g^−1^ biomass near the root apex to 3.5±0.8×10^9^ cells g^−1^ biomass at 160 mm from the root apex. The betaproteobacterial AOB specific 16S rRNA gene copy number was highest (2.3±1.8×10^8^ copies g^−1^ biomass) at ca. 20 mm from the root apex. *Nitrospira*-like NOB-specific 16S rRNA gene copy numbers were evenly distributed (10^7^ to 10^8^ copies g^−1^ biomass) from the root apex to 40 mm. The copy numbers of both AOB and *Nitrospira*-like NOB decreased markedly between 40 mm and 80 mm from the root apex and were under the detection limit (6.7×10^4^ and 4.0×10^4^ copies g^−1^ biomass, respectively) at 80 mm from the root apex. The *Nitrobacter*-like NOB-specific 16S rRNA gene copy numbers were one to two orders of magnitude lower (10^5^ to 10^6^ copies g^−1^ biomass) than those of *Nitrospira*-like NOB. The total bacterial cell count at the oxic sediment surface was 4.7±0.5×10^9^ cells g^−1^ sediment, which was comparable with the root biofilm. Both archaeal *amoA* gene and 16S rRNA gene were not amplified by PCR from the combined DNA samples (from 0 mm to 85 mm of the root), suggesting that the abundance of AOA in the root biofilm was under the detection limit.

### Community structure of AOB and NOB in the root biofilm

The 16S rRNA gene clone libraries were constructed from the biofilm attached to the root surface to investigate the betaproteobacterial AOB and *Nitrospira*-like NOB community structures. Forty-four clones were randomly selected from the betaproteobacterial AOB clone library, and the partial sequences of approximately 470 bp were analyzed. In total, 20 clone sequences were affiliated with the betaproteobacterial AOB and were grouped into 5 OTUs based on more than 97% sequence similarity among the OTUs. Their representative sequences were used for phylogenetic analysis (Fig. S2). We classified the betaproteobacterial AOB into seven stable lineages according to Purkhold *et al.*(38). The distribution of the 20 clones affiliated with known betaproteobacterial AOB is shown in Table 2. Twelve clones were related to the *Nitrosomonas oligotropha* lineage with 98.5–99.1% sequence similarity, seven clones were related to the *Nitrosomonas cryotolerans* lineage with 95.7–96.3% sequence similarity, and one clone was related to the *Nitrosomonas briensis* lineage with 95.7% sequence similarity.

Another 16S rRNA gene clone library was constructed from the root biofilm for the *Nitrospira*-like NOB community. Partial sequences of approximately 410 bp and 500 bp were analyzed from 47 and 23 clones retrieved using primer sets of NTSPAf-universal 1492r and Ntspa685-NTSPAr, respectively (Fig. S3). The distribution of all clones retrieved from the root biofilm using two different sets of PCR primers is shown in Table 3. The clones were mainly affiliated with the *Nitrospira moscoviensis* and *Nitrospira marina* lineages with more than 96.5% sequence similarity.

Microbial communities other than AOB and NOB in the root biofilm and bulk sediment were compared, showing higher microbial diversity in the root biofilm than in the bulk sediment (Table S1).

### Concentration profiles in the sediment

**Fig. 5:**
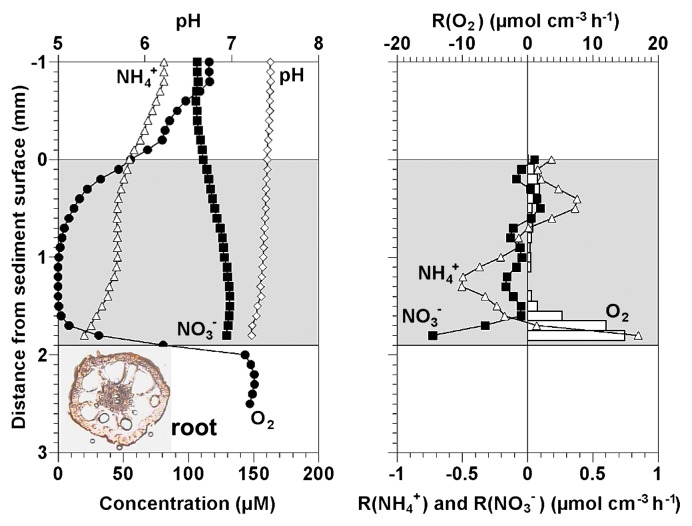
The representative steady-state concentration profiles of O_2_, NH_4_^+^, NO_3_^−^and pH (A) and the net volumetric consumption rates of O_2_, NH_4_^+^ and NO_3_^−^ (B) at ca. 25 mm from the root apex. Negative values indicate the production rates. Zero and 1.9 mm on the horizontal axis correspond to the sediment and root surfaces, respectively. Shaded area indicates the sediment.

The representative steady-state concentration profiles of O_2_, NH_4_^+^, NO_3_^−^ and pH in the sediment and rhizosphere of a *Phragmites* root at a position 25 mm from the root apex were measured with microelectrodes (Fig. 5A). O_2_ concentration was ca. 55 μM at the sediment surface (at 0 mm) and completely depleted at a depth of 1.1 mm from the sediment surface; however, O_2_ was detected again at ca. 0.5 mm above the root surface (at 1.5 mm from the sediment surface) and the O_2_ concentration increased up to ca. 80 μM at the root surface. The maximum O_2_ concentration inside the root was about 150 μM. NH_4_^+^ concentration decreased in the upper 0.5 mm of the oxic sediment surface whereas it was unchanged in the anoxic zone of the sediment (ca. 0.5 mm to 1.5 mm from the sediment surface). NH_4_^+^ concentration decreased again in the rhizosphere. NO_3_^−^ concentration gradually increased toward the depth of the sediment. pH slightly decreased in the rhizosphere.

Net volumetric consumption rates of O_2_, NH_4_^+^ and NO_3_^−^ were calculated from the concentration profiles (Fig. 5B). NH_4_^+^ consumption and production were observed in oxic and anoxic zones, respectively. The net NH_4_^+^ consumption rate determined from the NH_4_^+^ profile in the oxic rhizosphere was 0.023±0.007 μmol/cm^2^/h, which was higher than at the oxic sediment surface (0.016±0.006 μmol/cm^2^/h) (*p*<0.05). Net O_2_ consumption rate in the oxic rhizosphere (0.42±0.06 μmol/cm^2^/h) was also higher than that at the oxic sediment surface layer (0.085±0.004 μmol/cm^2^/h) (*p*<0.01).

Similarly, concentration profiles of O_2_, NH_4_^+^, and NO_3_^−^ were also measured at points ca. 5, 20, and 35 mm from the root apex, and net consumption and production rates of O_2_, NH_4_^+^, and NO_3_^−^ are summarized in Table 4. Net NH_4_^+^ and O_2_ consumption rates were high 20–25 mm from the root apex, which is consistent with the radial oxygen loss (ROL) (Figure 2) and abundance of AOB and NOB (Figure 4) along the root. We could not detect significant NH_4_^+^ consumption 50 mm from the root apex (data not shown).

## Discussion

### Radial oxygen release from roots

Several studies have shown that emergent plants such as *Phragmites*, *Scirpus validus*, and rice released O_2_ from their roots (4–7, 10, 40). Several analytical methods have been used to evaluate the radial oxygen release from roots. Among which, measurement using O_2_ microelectrodes provides high spatial (several 10 micrometers) and temporal resolution (4, 6, 7, 40). We demonstrated that O_2_ release was high in the apical region (up to ca. 40 mm from the root apex) and declined basipetally. The O_2_ release sufficiently supported the abundance and in situ activity of nitrifying bacteria in the biofilm attached to the roots of *Phragmites*.

Based on the O_2_ microprofiles that were determined in the anoxic sediment, the average radial oxygen loss (ROL) was determined to be 0.21 μmol O_2_ cm^−2^ root surface h^−1^ in this study, which was comparable with the previously reported values for *Phragmites* (0.15 μmol O_2_ cm^−2^ root surface h^−1^ at 25 mm from the root apex (5) and 0.11 μmol O_2_ cm^−2^-root surface h^−1^ at 7 mm from the root apex (4) and for *Scirpus validus* (0.113 μmol O_2_ cm^−2^ root surface h^−1^) (6). However, the oxic region only extended about 0.5 mm into the surrounding sediment (Figs. 3 and 5) due to a higher O_2_ consumption rate in the rhizosphere as compared with previous studies (4–6). Plant O_2_ release is highly dependent upon the plant (species, age, and health), sediment conditions (*e.g.*, oxidation reduction potential (ORP), biological and chemical oxygen demand, availability of nutrients, and other factors), and microbial activity in the rhizosphere (4–6).

### Abundance of nitrifying bacteria along the root

Very little is known about the in situ distribution of AOB, NOB, and AOA in the root biofilm. Cultivation-independent molecular analyses revealed that a mixed community of AOB and NOB was present in the biofilm attached to *Phragmits communis* roots and their levels of abundance were about one order of magnitude higher on a weight basis than the densities usually encountered in fresh water oxic sediment surfaces (28, 44) (Fig. 4).

A high abundance (10^7^ to 10^8^ copies g^−1^ biomass) of *Nitrosomonas*-like AOB and *Nitrospira*-like NOB was present in the apical region (up to ca. 40 mm from the root apex). The higher abundance in the apical region could be partly explained by higher oxygen release from the roots, which consequently resulted in the promotion of nitrification rates determined with microelectrodes (Fig. 5). Although the phenomenon of oxygen release by *Phragmites* roots has long been recognized, such oxygen release has never been convincingly linked to the ecology of AOB and NOB in the root-associated biofilm. Assuming that AOB and NOB have one 16S rRNA gene copy on average, since *N. europaea* NBRC 14298 carries one 16S rRNA gene copy (22), the betaproteobacterial AOB and *Nitrospira*-like NOB cell densities were approximately 10^7^ to 10^8^ cells g^−1^ biomass in the apical region (up to 40 mm from the root apex) of the root biofilm (Fig. 4). These values were comparable with the abundance of *Nitrosomonas* spp. in the root biofilm of rice (5.0–6.8×10^7^ cells cm^−3^ root) detected by FISH with an Nsm156 probe (7).

The 16S rRNA gene-cloning analysis for AOB shows that since the CTO primer used in this study were not completely specific to AOB of the *Betaproteobacteria*, several clone sequences were not affiliated with known AOB (Table 2). None of the published primers that are intended to target all AOB of the *Betaproteobacteria* show both 100% sensitivity (targeting all AOB) and 100% specificity (excluding all non-AOB) (9, 37). The same limitation inherent in PCR-based techniques also applies to the analysis of NOB in sediment and soil samples (Table 3). Thus, the possibility of underestimating and/or overestimating AOB- and NOB-specific 16S rRNA gene copy numbers cannot be excluded in this study; however, this does not negate the general trend that the abundance and activity of AOB and NOB was high in the apical region (up to ca. 40 mm from the root apex) due to high O_2_ release.

### Microbial community structure in the root biofilm

The results of 16S rRNA gene-cloning analysis revealed that the clones related to the *Nitrosomonas oligotropha* and *Nitrosomonas cryotolerans* lineages were numerically dominant in the root biofilms among AOB. *N. oligotropha* and *N. cryotolerans* have been isolated from freshwater and wastewater habitats and characterized by their ability to cope with low ammonia concentrations (*i.e.*, *K_m_* (NH_3_)=ca. 3 μM) (23, 36, 47). Although no kinetic data with respect to oxygen are available for *N. oligotropha*-like AOB, it could be speculated that they also have high affinity toward oxygen (17). Briones *et al.*(7) have shown that the community structure of root-associated AOB populations was dependent on the microscale O_2_ availability in the rhizosphere. For the NOB population, the clones related to *Nitrospira marina* and *Nitrospira moscoviensis* lineages dominated in the clone library. Dominance of *Nitrospira*-like NOB in freshwater sediment has been reported by several previous studies (1, 20, 28, 44). *N. moscoviensis* was originally isolated from the corroded iron pipe of a heating system (15). The *N. moscoviensis* lineage consists of uncultured bacterium clones retrieved from a non-chlorinated model drinking water distribution system (26) and from freshwater environmental metal-rich sediments from Green Bay (48). Inorganic and organic nitrogen concentrations in the overlaying water at the sampling site (n=25) were NH_4_^+^=not detected, NO_2_^−^=not detected, NO_3_^−^=193±11 μM, and total nitrogen 212±16 μM, which might explain the nitrifying bacterial communities in the root biofilm in this study.

Comparative investigation of the microbial community in the root biofilm and bulk sediment clearly showed higher microbial diversity in the root biofilm (Table S1). This is probably because the roots excrete organic matter as well as oxygen. This result clearly indicated that the oxic microenvironment developed near the immediate root environment by O_2_ leakage from the roots; otherwise, the anoxic bulk sediment prevailed.

### Potential contribution of an emergent plant

Changes in NH_4_^+^ and/or NO_3_^−^ concentrations in the immediate root environment could be attributed to microbial activities (nitrification, denitrification, and/or assimilation) and plant uptake (Fig. 5); however, we could not evaluate the relative contribution of each process to the overall net consumption of NH_4_^+^ and NO_3_^−^. Net NH_4_^+^ and O_2_ consumption rates at the root surface were 0.023±0.007 μmol NH_4_^+^ cm^−2^ h^−1^ and 0.42±0.06 μmol O_2_ cm^−2^ h^−1^, which were both higher than those at the oxic sediment surface (0.016±0.006 μmol NH_4_^+^ cm^−2^ h^−1^ and 0.085±0.004 μmol O_2_ cm^−2^ h^−1^). As a consequence, approximately 8% and 28% of O_2_ were used for ammonia oxidation at the root surface and the oxic sediment surface, respectively. This may suggest the occurrence of high competition for oxygen with heterotrophic bacteria, especially in the rhizosphere (30), because the roots excrete organic matter. Furthermore, oxygen could be used for oxidation of potential phytotoxins (*e.g.*, Fe^2+^, Mn^2+^, and sulfide) (52). Total NO_3_^−^ production rates at the root surface and at the oxic sediment surface (0.016±0.016 μmol NO_3_^−^cm^−2^ h^−1^ and 0.007±0.001 μmol NO_3_^−^ cm^−2^ h^−1^) were about 70% (0.016/0.023) and 40% (0.007/0.016) of the total NH_4_^+^ consumption rates, respectively. This might suggest that a proportion of NO_3_^−^ produced by nitrifying bacteria was taken up by the plant in the rhizosphere and/or simultaneously denitrified.

We evaluated the potential oxygen contribution of an emergent plant, *Phragmites*, to anoxic sediment at the sampling site (Sosei River, Sapporo, Japan). The amount of oxygen released by *Phragmites* roots per unit sediment surface area can be estimated based on the radial oxygen loss (ROL), the number and length of active roots, and the field root density, which was quantified at the sampling site. The total number of the roots was determined to be ca. 309±69 roots per 100 cm^−2^ -sediment surface. Assuming that only the 40 mm tip of a root of 0.5 mm average diameter can release O_2_ (Fig. 2), total O_2_ release (oxic) surface area of the root was then calculated to be 194 ± 44 cm^2^ root surface per 100 cm^−2^ sediment surface, indicating that about 2 times more oxic root surface area exists in the sediment. Based on oxygen microprofiles, the average amount of O_2_ released by the *Phragmites*, namely ROL, was calculated to be 0.21 μmol O_2_ cm^−2^ root surface h^−1^ (Fig. 2); therefore, we now can calculate the amount of oxygen released by *Phragmites* roots per 100 cm^2^ of the sediment surface to be 0.21×194=40.7 μmol O_2_ per 100 cm^−2^ sediment surface h^−1^, corresponding to 3.14 g-O_2_ m^−2^ sediment surface d^−1^. This value is within the range of the amount of oxygen released by wetland plants reported by various researchers (summarized by Bezbaruah and Zhang (6)). This result indicated that *Phragmites* roots played a significant role in the oxygen supply to freshwater sediments.

In conclusion, we successfully determined oxygen release by *Phragmites* roots using a oxygen microelectrode and then related this to the in situ abundance and activity of AOB and NOB in the rhizosphere environment by the combined use of 16S rRNA gene-cloning analysis, qPCR assay and microelectrodes. O_2_ was released from the apical region (up to ca. 40 mm from the root apex) with a high abundance of nitrifying bacteria (10^7^ to 10^8^ copies g^−1^-biomass). The total NH_4_^+^ and O_2_ consumption rates in the apical region were higher than those at the oxic sediment surface. These results suggest that nitrifying bacteria associated with *Phragmites* roots played an important role in NH_4_^+^ consumption in the waterlogged anoxic sediment.

## Supplementary Material



## Figures and Tables

**Table 1:** Net consumption and production rates of NH_4_^+^, NO_2_^−^, and NO_3_^−^ in the horizontal flow reactors with and without *Phragmites*^a^

Net consumption rate (μmol cm^−2^ sediment surface h^−1^)	with *Phragmites*	without *Phragmites*
NH_4_^+^	0.039±0.016	0.020±0.007
NO_2_^−^	0.010±0.007	−0.006±0.010
NO_3_^−^	−0.037±0.019	−0.036±0.015

a Negative values indicate the production rates. The rates were calculated from the influent and effluent concentrations of NH_4_^+^, NO_2_^−^, and NO_3_^−^ (n=53).

**Table 2:** Detection frequency and phylogenetic relatives of the AOB clones obtained from rhizosphere

Lineage and Closest relative	No. of OTUs (No. of clones)	Similarity
Total	44	
*Nitrosomonas oligotropha* lineage		
*Nitrosomonas* sp. Is79A3 (AJ621026)	2 (12)	98.5–99.1
*Nitrosomonas cryotolerans* lineage		
*Nitrosomonas cryotolerans* (AF272423)	2 (7)	95.7–96.3
*Nitrosomonas briensis* lineage		
*Nitrosospira* sp. 9SS (EF015570)	1 (1)	95.7

**Table 3:** Detection frequency and phylogenetic relatives of *Nitrospira*-like NOB clones obtained from rhizosphere

Lineage and Closest relative	No. of OTUs (No. of clones)		Similarity

	NTSPAf-1492r	NTSPA685-NTSPAr	
Total	47	23	
*Nitrospira defluvii* lineage			
*Nitrospira* sp. strain RC99 (Y14643)		1 (1)	100
*Nitrospira moscoviensis* lineage			
*Nitrospira sp.* (Y14644)	4 (14)		96.5–98.0
*Nitrospira sp.* (AF035813)	1 (2)		98.3
*Nitrospira* cf. *moscoviensis* SBR1024 (AF155153)		1 (1)	97.3
*Nitrospira* sp. (AF035813)		1 (1)	97.5
*Nitrospira marina* lineage			
*Nitrospira marina* Nb-295 (X82559)		2 (20)	97.6–97.8

**Table 4:** Net consumption rates of O_2_, NH_4_^+^, and NO_3_^−^ along the root. Negative values indicate the production rates

Net rate (μmol cm^−3^ h^−1^)	Approximate distance from root apex (mm)			
	5	20	25	35
O_2_	0.019	0.113	0.420	0.028
NH_4_^+^	0.002	0.013	0.023	0.002
NO_3_^−^	−0.009	−0.017	0.003	−0.010
