# Supplementary material for: Community Structure and In Situ Activity of Nitrifying Bacteria in Phragmites Root-Associated Biofilms
Source: Microbes Environ. 2012 Mar 23;27(3):242–9. doi: 10.1264/jsme2.ME11314 (PMC4036055; doi:10.1264/jsme2.ME11314)
Supplement: Supplementary file 1 [file 27_242_s1.pdf]

Fig. S1. A photograph of a thin cross section of a *Phragmites* root with brown thin biofilm.

Fig. S2. Phylogenetic tree showing the distribution of OTUs related to betaproteobacterial AOB. The tree was generated using approximately 470 bp of the 16S rRNA genes and the neighbor-joining method. Scale bar represents 2% sequence divergence. The values at the nodes are bootstrap values (1,000 resampling analysis). The *Nitrospira marina* sequence served as the outgroup for rooting the tree. Numbers in parentheses indicate the frequency of the appearance of identical clones in the clones analyzed.

Fig. S3. Phylogenetic trees showing the distributions of the OTUs related to *Nitrospira*-like NOB. The 16S rRNA gene fragments were amplified by using the *Nitrospira*-like NOB specific primer sets of NTSPAf (28) -universal 1492r (51) (A) and Ntspa685 (20) -NTSPAr (28) (B). The trees were generated by using approximately 410 bp (A) and 500 bp (B) of the 16S rRNA genes and the neighbor-joining method. Scale bar represents 2% sequence divergence. Values at the nodes are bootstrap values (1,000 resampling analysis). The *Nitrosomonas europaea* sequence served as the outgroup for rooting the tree. Numbers in parentheses indicate the frequency of the appearance of identical clones in the clones analyzed.

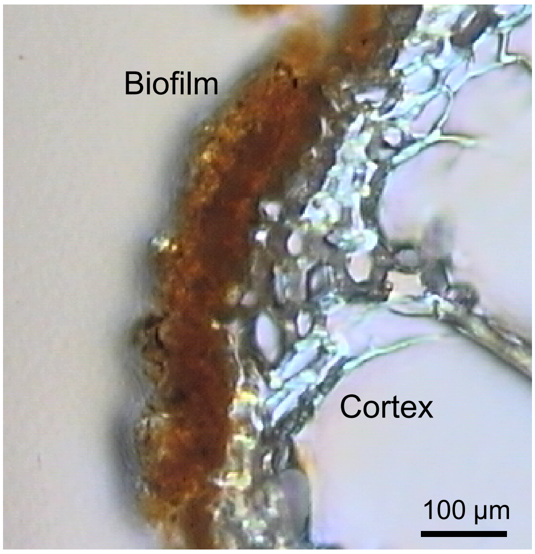

Supplemental material
Figure S1. Okabe *et al.*,

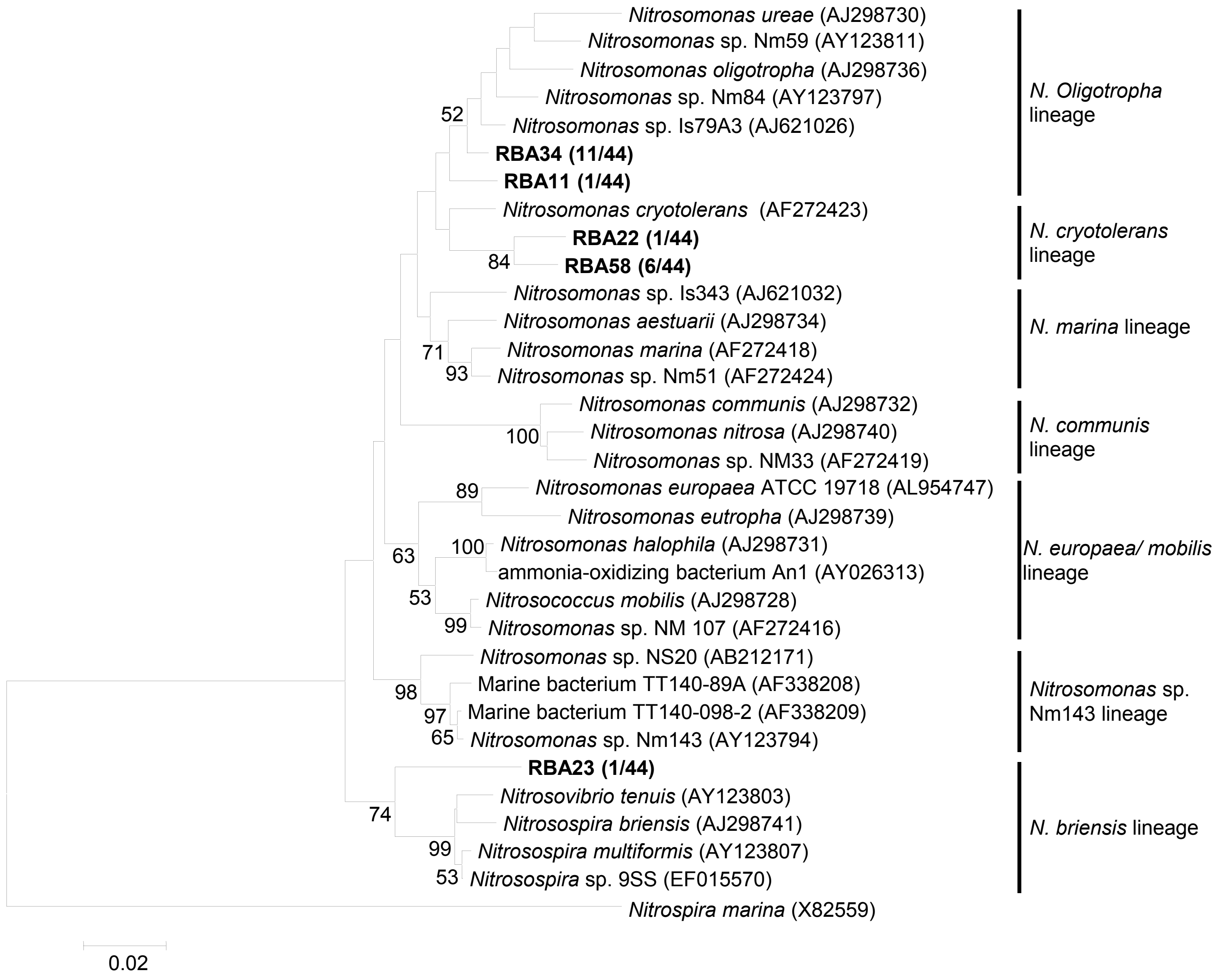

Supplemental material
Figure S2. Okabe *et al.*,

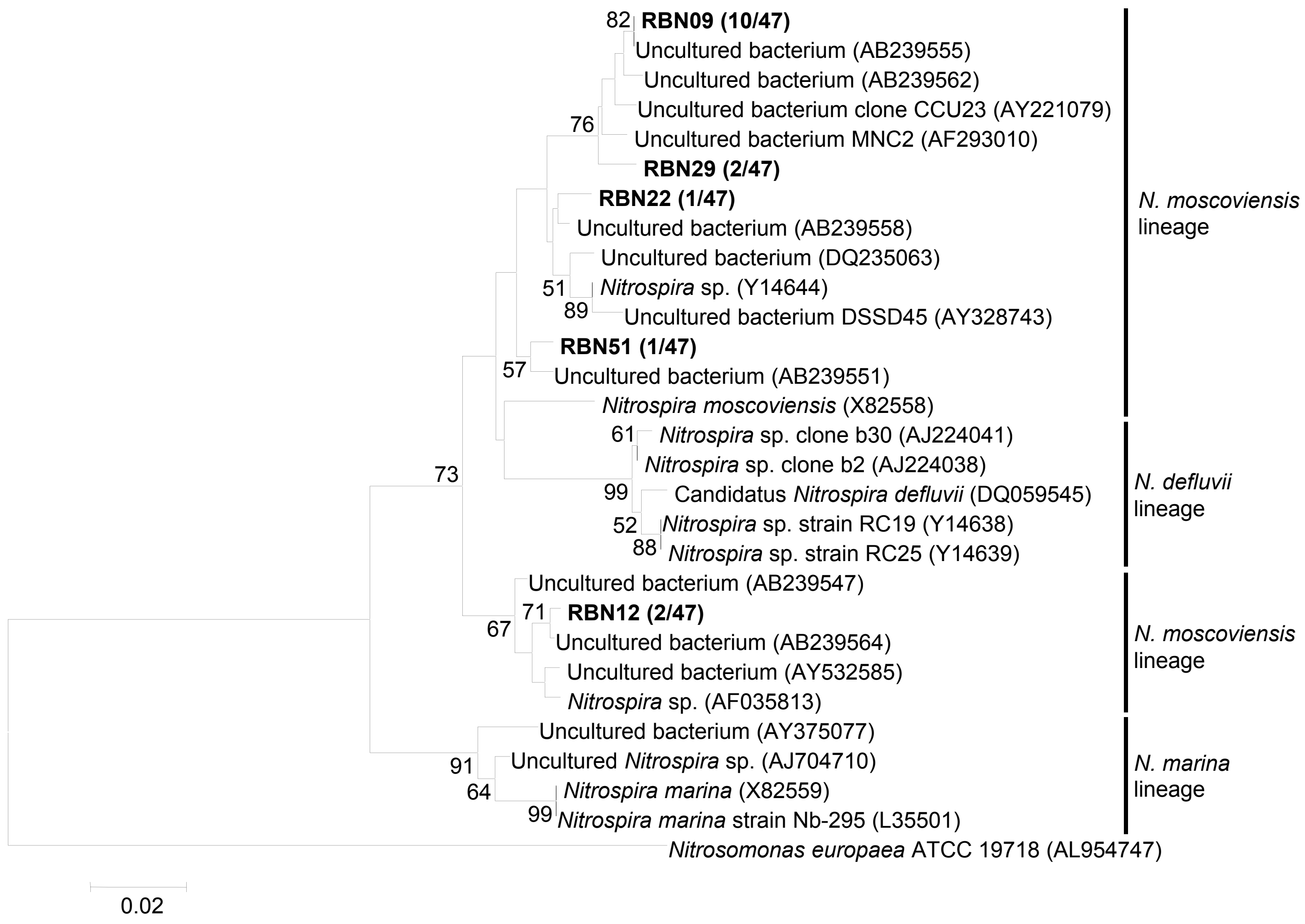

Supplemental material

Figure S3A. Okabe *et al.*,

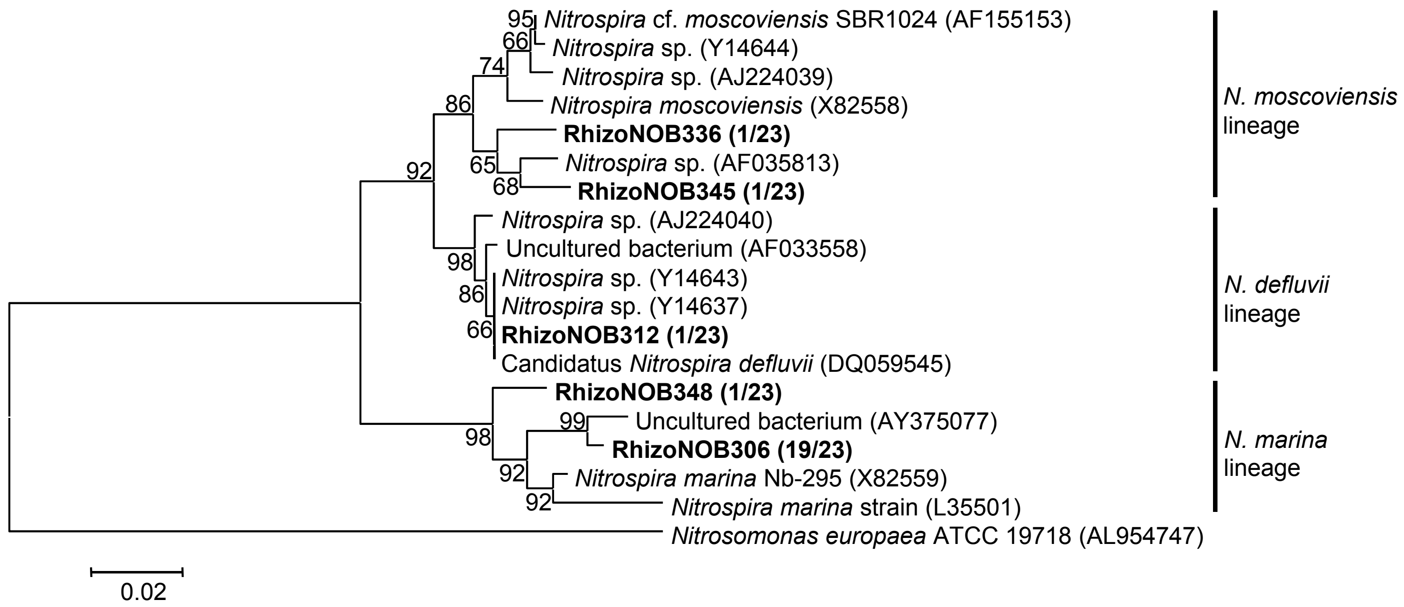

Supplemental material

Figure S3B. Okabe *et al.*,

TABLE S1. 16S rRNA gene phylotype distributions in the rhizosphere (root biofilm) and bulk sediment

Taxon	Genus	No. of OTUs (No. of clones) in the following samples	
		Bulk soil	Rhizospher
Total		54 (131)	98 (155)
Acidobacteria	*Geothrix*	1 (1)	1 (1)
	Unclassified	14 (36)	8 (9)
Actinobacterium	*Agromyces*	1 (1)	
	Arthrobacter	1 (1)	
	Nocardioides		1 (1)
	Terrabacter	1 (1)	
	Unclassified	1 (1)	1 (2)
Bacilli	*Bacillus*		1 (1)
Clostridia	*Acetivibrio*		1 (1)
	Clostridium	2 (3)	2 (5)
	Geosinus		1 (3)
	Unclassified	1 (1)	3 (6)
Bacteroidetes	*Cytophaga*	1 (1)	
	Flavobacterium	1 (23)	2 (4)
	Prolixibacter	1 (1)	1 (1)
	Unclassified	4 (6)	6 (11)
Alphaproteobacteria	*Acidosphaera*	1 (1)	
	Bradyrhizobium		1 (2)
	Labrys	1 (1)	1 (1)
	Mesorhizobium		1 (2)
	Methylocapsa	1 (1)	
	Rhizobium		1 (1)
	Skermanella	1 (1)	
	Unclassified	1 (1)	1 (5)
Betaproteobacteria	*Acidovorax*		1 (1)
	Aquamonas		1 (4)
	Aquaspirillum		2 (2)
	Azospira		1 (3)
	Curvibacter		1 (1)
	Dechloromonas		1 (3)
	Gallionella		1 (1)
	Ideonella		1 (1)
	Methylibium		1 (3)
	Methylophilus		1 (3)
	Nitrosomonas		1 (3)
	Nitrosospira	1 (1)	1 (1)
	Psedomonas		2 (3)
	Ramlibacter	1 (6)	1 (1)
	Rhodocyclus		1 (5)
	Rubrivivax		1 (2)
	Thiobacillus		
	Thiobacter		1 (1)
	Sterolobacterium		1 (4)
	Unclassified	2 (6)	4 (5)

Continued on next page

TABLE S1—*Continued*

Taxon	Genus	No. of OTUs (No. of clones) in the following samples	
		Bulk soil	Rhizospher
Deltaproteobacteria	*Anaeromyxobacter*	2 (11)	
	Desulfobulbus		1 (1)
	Geobacter	2 (2)	2 (4)
	Unclassified	1 (1)	7 (7)
Gammaproteobacteria	*Aquimonas*		1 (1)
	Methylobacter		1 (2)
	Methylomonas		1 (1)
	Nitrococcus		1 (1)
Chloroflexi	*Anaerolinea*		1 (1)
	Dehalococcoides	2 (2)	1 (1)
	Kouleothrix		1 (1)
	Unclassified	1 (1)	6 (7)
Nitrospirae	*Magnetobacterium*		2 (2)
	Nitrospira		1 (1)
	Unclassified		1 (1)
Planctomycetes	*Gemmate*		1 (1)
	Pirellula		1 (1)
	Unclassified	2 (2)	
Verrucomicrobia	*Opitutus*		1 (1)
	Verrucomicrobium		1 (1)
	Xiphinematobacter		1 (1)
	Unclassified		2 (2)
Cyanobacteria	Unclassified		1 (2)
Gemmatimonadetes	Unclassified	5 (8)	5 (5)
OP10	Unclassified		3 (3)
Spirochaetes	*Spirochaeta*		1 (1)
Unclassified	Unclassified	1 (9)	5 (5)
